# Evaluation of the introduction of novel potassium binders in routine care; the Stockholm CREAtinine measurements (SCREAM) project

**DOI:** 10.1007/s40620-023-01860-0

**Published:** 2024-01-18

**Authors:** Ailema Gonzalez-Ortiz, Catherine M. Clase, Alessandro Bosi, Edouard L. Fu, Beatriz E. Pérez-Guillé, Anne-Laure Faucon, Marie Evans, Carmine Zoccali, Juan-Jesús Carrero

**Affiliations:** 1 Department of Medical Epidemiology and Biostatistics, Karolinska Institutet, Nobels Väg 12A, Box 281, 171 77 Stockholm, Solna, Sweden; 2 Translational Research Center, Instituto Nacional de Pediatría, Mexico City, Mexico; 3 Department of Medicine, McMaster University, Hamilton, ON Canada; 4 Department of Health Research and Methodology, McMaster University, Hamilton, ON Canada; 5 Division of Pharmacoepidemiology and Pharmacoeconomics, Department of Medicine, Brigham and Women’s Hospital, Harvard Medical School, Boston, MA USA; 6 Department of Clinical Epidemiology, Leiden University Medical Center, Leiden, The Netherlands; 7 INSERM U1018, Department of Clinical Epidemiology, Centre for Epidemiology and Population Health, Paris-Saclay University, Gif-sur-Yvette, France; 8 Department of Clinical Science, Intervention and Technology, Karolinska Institutet, Stockholm, Sweden; 9 CNR-IFC, Clinical Epidemiology of Renal Diseases and Hypertension, Reggio Calabria, Italy; 10 Department of Clinical Sciences, Karolinska Institutet, Danderyd Hospital, Stockholm, Sweden

**Keywords:** Patiromer, Sodium zirconium cyclosilicate, Sodium polystyrene sulfonate, Hyperkalemia, SCREAM

## Abstract

**Background:**

The pharmacological management of hyperkalemia traditionally considered calcium or sodium polystyrene sulfonate and, since recently, the novel binders patiromer and sodium zirconium cyclosilicate. We evaluated their patterns of use, duration of treatment and relative effectiveness/safety in Swedish routine care.

**Methods:**

Observational study of adults initiating therapy with sodium polystyrene sulfonate or a novel binder (sodium zirconium cyclosilicate or patiromer) in Stockholm 2019–2021. We quantified treatment duration by repeated dispensations, compared mean achieved potassium concentration within 60 days, and potential adverse events between treatments.

**Results:**

A total of 1879 adults started treatment with sodium polystyrene sulfonate, and 147 with novel binders (*n* = 41 patiromer and *n* = 106 sodium zirconium cyclosilicate). Potassium at baseline for all treatments was 5.7 mmol/L. Sodium polystyrene sulfonate patients stayed on treatment a mean of 61 days (14% filled ≥3 consecutive prescriptions) compared to 109 days on treatment (49% filled ≥3 prescriptions) for novel binders. After 15 days of treatment, potassium similarly decreased to 4.6 (SD 0.6) and 4.8 (SD 0.6) mmol/L in the sodium polystyrene sulfonate and novel binder groups, respectively, and was maintained over the 60 days post-treatment. In multivariable regression, the odds ratio for novel binders (vs sodium polystyrene sulfonate) in reaching potassium ≤ 5.0 mmol/L after 15 days was 0.65 (95% CI 0.38–1.10) and after 60 days 0.89 (95% CI 0.45–1.76). Hypocalcemia, hypokalemia, and initiation of anti-diarrheal/constipation medications were the most-commonly detected adverse events. In multivariable analyses, the OR for these events did not differ between groups.

**Conclusion:**

We observed similar short-term effectiveness and safety for all potassium binders. However, treatment duration was longer for novel binders than for sodium polystyrene sulfonate.

**Graphical abstract:**

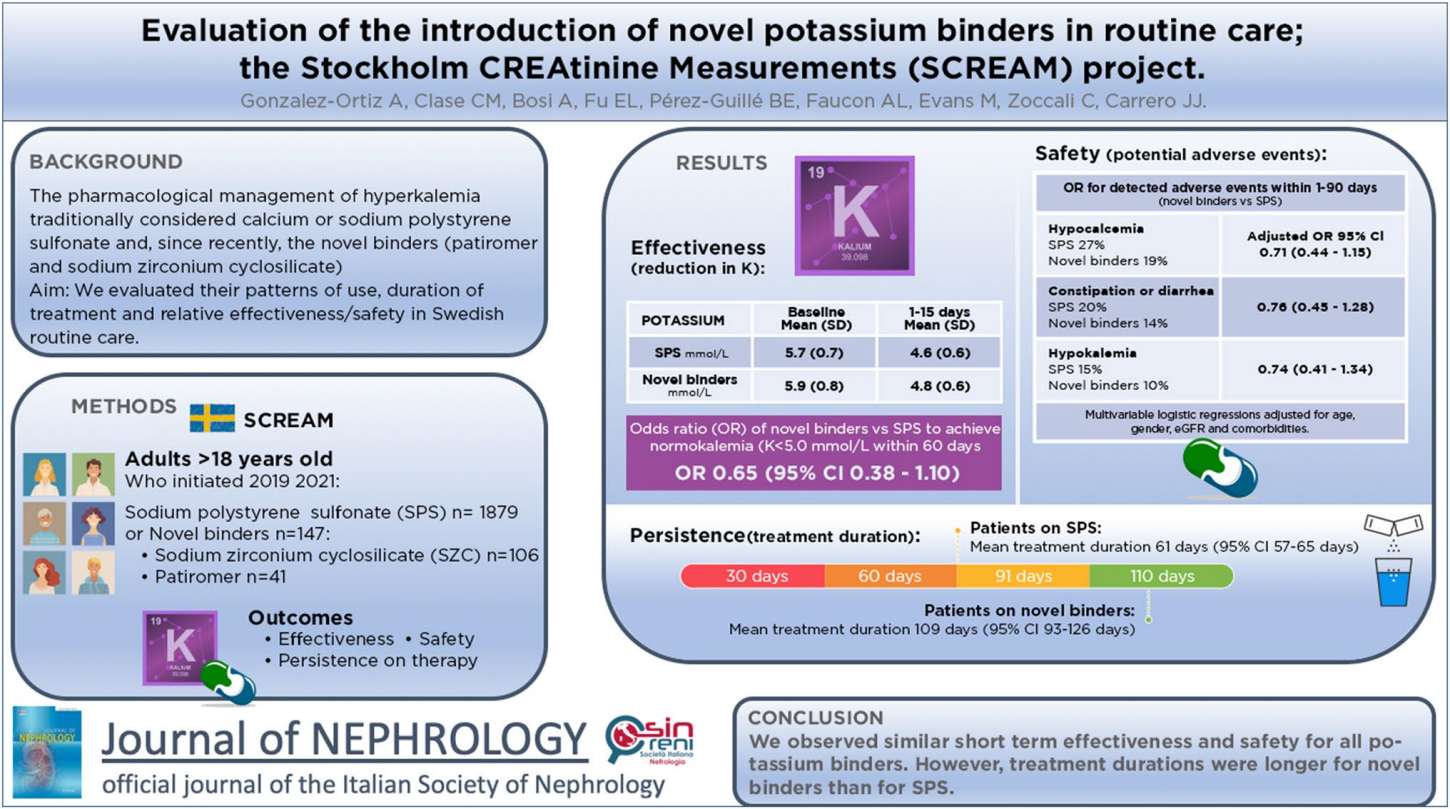

**Supplementary Information:**

The online version contains supplementary material available at 10.1007/s40620-023-01860-0.

## Introduction

Hyperkalemia is a common and potentially fatal electrolyte abnormality attributed to excess dietary potassium intake, comorbidity burden, metabolic disorders, or intake of medications that interfere with potassium excretion [1, 2]. These conditions often coincide in people with chronic kidney disease (CKD), compounding the risk of hyperkalemia.

Sodium and calcium polystyrene sulfonate have been available for 70 years for the management of hyperkalemia. Recently, two additional novel cation exchangers, patiromer sorbitex calcium (patiromer) and sodium zirconium cyclosilicate, received regulatory approval. All agents have shown short- and mid-term efficacy in clinical trials treating acute hyperkalemia against the non-pharmacological standard-of-care [3–9]. No trial to date has performed head-to-head comparisons of all three agents. However, a small randomized cross-over trial in patients on dialysis recently suggested sodium polystyrene sulfonate to be more effective than patiromer in reducing mean weekly potassium [10]. In their pivotal trials, all agents consistently reported the occurrence of minor gastrointestinal disorders and low electrolyte levels [3–9, 11]. A network meta-analysis of these trials[12] suggested a similar rate of minor adverse effects across all three agents and a higher probability of nausea and constipation among patiromer users compared to users of sodium zirconium cyclosilicate or sodium polystyrene sulfonate.

Clinical trials are carefully conducted experiments with strict monitoring routines in highly selected patients. Phase IV post-marketing studies are needed to confirm safety and efficacy in the heterogeneous clinical practice, where therapies are given to patients with higher levels of comorbidity and where there is usually greater variability in clinical monitoring or compliance with therapy. Some observational studies have been published to date, evaluating the introduction of single potassium binders.[13–21]. Currently, no routine-care studies have compared all three agents.

Healthcare workers in Sweden have prescribed sodium polystyrene sulfonate routinely for decades; sodium zirconium cyclosilicate and patiromer became available in Sweden in late 2018. In this study, we describe the treatment practice of potassium binders after the introduction of the novel binders and compare the use, safety, and effectiveness of all three agents in contemporary routine care in a single-provider health-care system in Stockholm.

## Materials and methods

### Data sources

The study population is derived from the Stockholm CREAtinine Measurements (SCREAM) project, a health care utilization cohort that includes all citizens of the region of Stockholm, Sweden [22]. Laboratory data were linked with regional and national administrative databases for complete information on healthcare utilization, diagnoses and procedures, dispensed drugs, and follow-up. The Stockholm Ethics Review Board approved the study with a waiver of consent.

### Study population

For this study, we included all adults (> 18 years) who newly initiated sodium polystyrene sulfonate, sodium zirconium cyclosilicate, or patiromer during 2019–2021. New initiations were defined as a pharmacy dispensation with at least 6 preceding months without any other recorded potassium binder use. The date of the first dispensation was the index date (baseline), at which point all covariates were assessed, and follow-up started. We followed patients for up to 365 days or until death, emigration from the region, or end of data collection, whichever occurred first.

### Study exposure

The study exposures were the different potassium binders started, identified by filled dispensations in Swedish pharmacies with the following ATC codes: V03AE01 (for sodium polystyrene sulfonate), V03AE09 (patiromer), and V03AE10 (sodium zirconium cyclosilicate). Calcium polystyrene sulfonate is not commercialized in Sweden. The indication for sodium polystyrene sulfonate in Sweden is to treat hyperkalemia in subjects with CKD; however, there is a long-standing clinical practice of using low doses of sodium polystyrene sulfonate for hyperkalemia prevention in patients with a history of elevated potassium. [23] Both patiromer and sodium zirconium cyclosilicate are approved in Sweden for treating hyperkalemia in adults, but costs are subsidized by the Swedish Government only when prescribed to patients with heart failure or patients with CKD G3 to 5 for whom treatment with sodium polystyrene sulfonate is not appropriate/sufficient/tolerated. [24]

### Study covariates

We calculated study covariates at the index date, extracting data on age, gender, laboratory tests, comorbidities, and medications. Comorbidities were defined by the presence of relevant diagnostic codes [International Classification of Diseases (ICD)] prior to index date (Supplementary Table 1). Medications were assumed concomitant if there was a pharmacy dispensation at the time of, or within, the previous 6 months from the index date (Supplementary Table 2). Out-patient plasma creatinine was used to estimate glomerular filtration rate (eGFR) with the 2009 Chronic Kidney Disease Epidemiology Collaboration (CKD-EPI) equation [25] without adjusting for race; Swedish law prohibits collection of race information. Chronic kidney disease was categorized into KDIGO categories [26] as follows: ≥ 60 ml/min/1.73 m^2^, 45–59 ml/min/1.73 m^2^, 30–44 ml/min/1.73 m^2^; 15–29 ml/min/1.73 m^2^, and < 15 ml/min/1.73 m^2^ including those undergoing kidney replacement therapy (KRT). Plasma and serum potassium levels from all types of care, in-patient and out-patient, were extracted during the 90 days before the index date to evaluate indications for therapy. The highest potassium within that period was categorized into four mutually exclusive groups: normokalemia (≤ 5.0 mmol/L), mild (5.1–5.49 mmol/L), moderate (5.5–5.9 mmol/L) and severe hyperkalemia (≥ 6.0 mmol/L).

### Study outcomes

#### Persistence on therapy

The duration of treatment was ascertained during the 365 days after initiation by consecutive fills and the time (in days) between them. The expected duration of the fill (i.e., the number of days that one package of medication was expected to cover) was that stipulated in the product label, which tended to be around 30 days for all three agents. Discontinuation of therapy was defined by the absence of a new dispensation during additional 30 days after the end of the last estimated pill supply.

#### Achieved potassium concentrations

There is no consensus recommendation for the frequency of potassium monitoring [1, 27, 28], and in routine care, this varies by patient and practitioner. As in previous studies [14, 16], we evaluated mean achieved potassium levels, if measured, and within the 0–15 days, 16–30 days, 31–45 days, and 46–60 days after treatment initiation. For each period, we considered all patients with at least one potassium measurement. In our primary analyses, we modeled the potassium value closest to the end of each time interval. As a sensitivity analysis, we averaged all the potassium values per patient during each follow-up interval.

#### Evaluation of potential adverse events

Likewise, there is no consensus recommendation for the frequency of monitoring for adverse events, and in routine care this also varies. We explored the occurrence of adverse events potentially associated with potassium binder use, as reported in their product labels, within the first 90 days of therapy. These included (definitions detailed in Supplementary Table 3): severe adverse gastrointestinal events (intestinal ischemia or thrombosis, gastrointestinal ulcers, and perforation); minor adverse gastrointestinal events (de novo dispensation of laxatives or anti-diarrheal drugs among those free from those medications at baseline)[23]; abnormal plasma electrolyte levels: hypokalemia (potassium < 3.5 mmol/L), hypophosphatemia (phosphate < 0.81 mmol/L), hypocalcemia (total calcium < 2.15 mmol/L) and hypernatremia (sodium > 145 mmol/L). [29–31].

### Statistical analysis

Values are expressed as mean and standard deviation (SD) for continuous variables with normal distribution, median (interquartile range, IQR) for non-normal distribution variables and percentage of total for categorical. We studied all novel potassium binders as a group: because of small sample sizes, we were not able to study individual novel potassium binders. We constructed survival curves using the Kaplan–Meier method to show persistence on therapy over time. We compared mean potassium concentration at the different time intervals against pre-treatment potassium with paired t tests or one-way ANOVA and evaluated the difference between proportions of patients with potassium > 5.0 and > 5.5 mmol/L with the Chi square test. Serum potassium variability over the first 60 days after treatment start was assessed as the coefficient of variation (CV; standard deviation/mean) of all available tests. We used logistic regression to calculate a) the odds ratio (OR) and 95% confidence interval (CI) of novel binders for achieving normokalemia (≤ 5.0 mmol/L) during treatment compared to sodium polystyrene sulfonate; and b) the OR of novel binders for the presence of potential adverse events at fixed time intervals compared to sodium polystyrene sulfonate. As a sensitivity analysis, we repeated analyses using the mean of all the potassium values per patient during each follow-up interval. All statistical analyses were conducted using R version 3.5.1 and STATA software (version 17.1; Stata Corp, College Station, TX).

## Results

In the Stockholm region during 2019–2021, 2130 subjects initiated potassium binders. After excluding 83 patients younger than 18 years old and 21 who were not residents of Stockholm, the study population consisted of 2026 participants, 33% women, with a mean age of 68 (SD 16) years. Of those, 1879 initiated treatment with sodium polystyrene sulfonate, and 147 with novel binders (41 with patiromer and 106 with sodium zirconium cyclosilicate).

Baseline characteristics by treatment are presented in Table 1. New users of sodium polystyrene sulfonate had a mean age of 68 (SD 16) years, mean eGFR 34 (SD 20) ml/min/1.73 m^2^, and 32% were women. New users of novel binders had a mean age of 63 (SD 17) years, mean eGFR 36 (SD 21) ml/min/1.73 m^2^, and 44% were women. Hypertension *n* = 1768 (87%), diabetes *n* = 918 (45%), and heart failure *n* = 683 (34%) were the three most common comorbidities in both groups, with common use of angiotensin converting enzyme/angiotensin receptor blockers, beta-blockers and diuretics. A history of hyperkalemia or previous history of sodium polystyrene sulfonate use was more common among novel binder initiators. Patients starting patiromer were older and had a higher eGFR and a larger proportion of baseline heart failure than patients on sodium zirconium cyclosilicate. Median potassium concentration at the start of treatment was 5.7 mmol/L, similar across therapies (5.7 mmol/L in the sodium polystyrene sulfonate group and 5.8 mmol/L in the novel binder group). The last potassium value before treatment initiation was < 5.0 in 15% of patients starting sodium polystyrene sulfonate and 9% in patients starting novel binders.

**Table 1:** Baseline characteristics of study participants by treatment initiated

Variable	SPS	Novel potassium binders	Patiromer	SZC
*n*	1879	147	41	106
Age (in years) mean, (SD)	68 (16)	63 (17)	68 (14)	61 (17)
Women *n*, (%)	596 (32)	65 (44)	17 (42)	48 (45)
eGFR (in ml/min/1.73 m^2^) mean, (SD)	34 (20)	36 (21)	52 (18)	29 (19)
CKD severity categories				
CKD G1-G2 *n*, (%)	149 (8)	16 (11)	11 (27)	5 (5)
CKD G3 *n*, (%)	474 (25)	32 (22)	17 (41)	15 (15)
CKD G4 *n*, (%)	399 (21)	27 (18)	1 (2)	26 (25)
CKD G5 *n*, (%)	213 (12)	17 (12)	2 (5)	15 (14))
KRT *n*, (%)	532 (28)	40 (27)	3 (7)	37(35)
Unknown *n*, (%)	112 (6)	15 (10)	7 (17)	8 (8)
Hypertension *n*, (%)	1647 (88)	121 (82)	32 (78)	89 (84)
Diabetes Mellitus *n*, (%)	853 (45)	65 (44)	16 (39)	49 (46)
Heart failure *n*, (%)	623 (33)	60 (41)	26 (63)	34 (32)
Myocardial infarction *n*, (%)	265 (14)	18 (12)	7 (17)	11 (10)
Arrhythmia *n*, (%)	316 (16)	25 (17)	9 (22)	16 (15)
Inflammatory bowel disease *n*, (%)	85 (5)	9 (6)	1 (2)	8 (8)
History of hyperkalemia (by diagnosis) *n, (%)	476 (25)	46 (31)	10 (24)	36 (34)
History of SPS use in the previous year, n, (%)	495 (26)	61 (41)	8 (19)	53 (50)
Betablockers *n*, (%)	1217 (65)	95 (65)	30 (73)	65 (61)
Thiazide or loop diuretics *n*, (%)	999 (53)	83 (57)	25 (61)	58 (55)
ACE inhibitors *n*, (%)	633 (34)	51 (35)	14 (34)	37 (35)
Angiotensin Receptor Blockers n, (%)	679 (36)	72 (49)	26 (63)	46 (43)
Mineralocorticoid Receptor Antagonists *n*, (%)	172 (9)	30 (20)	18 (44)	12 (11)
Antiplatelets/Aspirin *n*, (%)	674 (36)	42 (29)	15 (37)	27 (26)
Proton Pump Inhibitors *n*, (%)	824 (44)	65 (44)	14 (34)	51 (48)
Potassium tests in the 90 days before dispensation				
Number of patients with potassium test *n*, (%)	1661 (88)	127 (86)	31 (76)	96 (91)
Number of potassium tests per patient, median (IQR) mean (SD)	6 (3–15) 13 (18)	7 (4–15) 12 (16)	4 (3–6) 6 (5)	8 (4–17) 14 (18)
Highest potassium (mmol/L), median (IQR) mean (SD)	5.7 (5–6) 5.7 (0.7)	5.7 (5–6) 5.9 (0.8)	5.6 (5–6) 5.7 (0.6)	5.7 (5–6) 5.9 (0.9)
Potassium categories				
No values in previous 90 days *n*, (%)	218 (12)	20 (14)	10 (24)	10 (9)
No hyperkalemia (≤ 5.0 mmol/L) *n*, (%)	248 (13)	12 (9)	2 (5)	10 (9)
Mild hyperkalemia (5.1–5.49 mmol/L) *n*, (%)	339 (18)	28 (22)	7 (17)	21 (20)
Moderate hyperkalemia (5.5–5.9 mmol/L) *n*, (%)	522 (28)	44 (35)	13 (32)	31 (29)
Severe hyperkalemia (≥ 6 mmol/L) *n*, (%)	552 (29)	43 (34)	9 (22)	34 (32)

^*^Hyperkalemia history ICD diagnosis (E875)

*SPS* sodium polystyrene sulfonate, *SZC* sodium zirconium cyclosilicate, *eGFR* estimated GFR by the 2009 CKD-EPI equation without adjustment for race, *CKD* chronic kidney disease, *ACE* angiotensin converting enzyme, *KRT* kidney replacement therapy

**Fig. 1:**
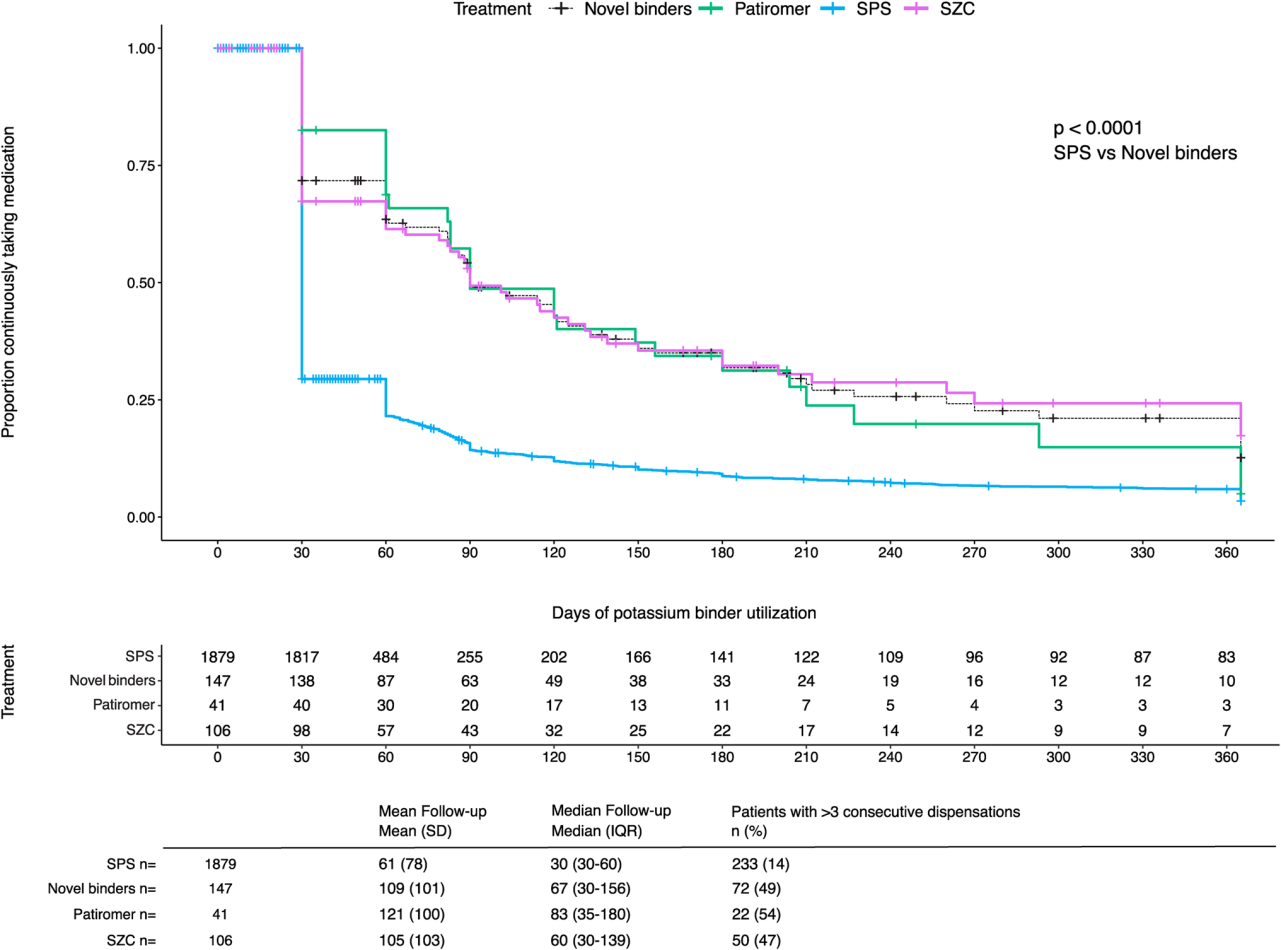
Duration of continuous therapy after initiation of potassium binders up to 365 days after treatment initiation by binder type. Legend: Persistence on therapy was estimated based on repeated dispensations for each agent (see methods). *P* value denotes statistically significant difference in the persistence of SPS vs novel potassium binders, using the log-rank test

Treatment duration was longer for novel binders than sodium polystyrene sulfonate (Fig. 1). Patients on sodium polystyrene sulfonate stayed on treatment for a mean of 61 days, and 14% filled three or more consecutive prescriptions suggesting chronic use. Patients on novel binders stayed on treatment for a mean of 109 days, and 49% filled three or more prescriptions.

**Fig. 2:**
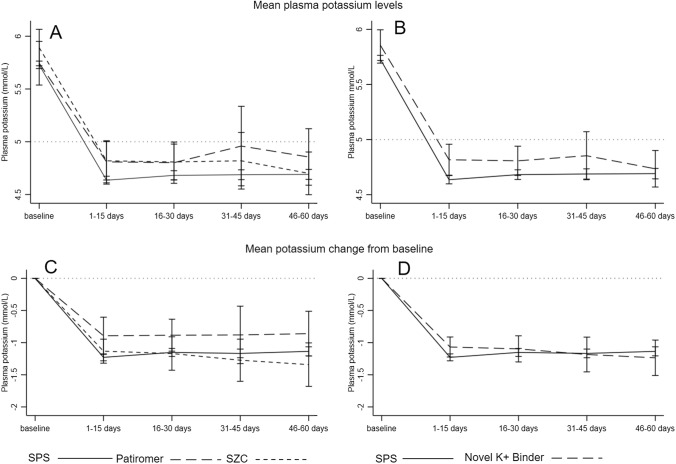
Mean potassium concentration during different intervals after treatment start (panels **A** and **B**) and mean potassium change from baseline potassium (panels **C** and **D**). Legend: Shown is the mean potassium value closest to the end of each time interval. Panels **A** and **C** show each agent separately (SPS, patiromer and SZC), and panels **B** and **D** combine both novel potassium binders together. There were no statistically significant differences between treatment strategies (*P* > 0.05) as estimated by t-test or one-way ANOVA. SPS; sodium polystyrene sulfonate, SZC; sodium zirconium cyclosilicate, Novel K + ; Binder (SZC + patiromer)

**Fig. 3:**
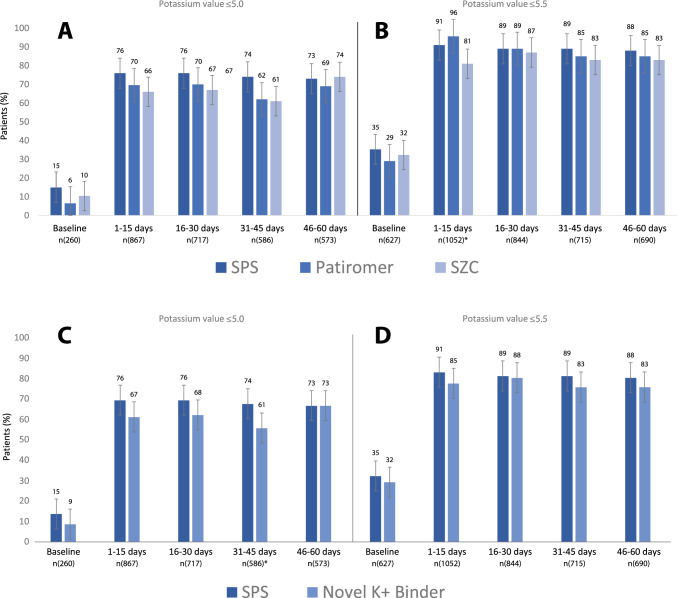
Proportion of patients with plasma potassium concentration ≤ 5.0 mmol/L (Panels **A** and **C**) and ≤ 5.5 mmol/L (Panels **B** and **D**) before and after initiation of treatment with potassium binders. Legend: Bars (standard error) represent the percentage of patients with potassium ≤ 5.0 mmol/L and ≤ 5.5 mmol/L, modeling the potassium value closest to the end of each time interval. N () shows the number of patients represented in each of the bars. *There were no statistically significant differences between the binders, except for one observation in panel B during the first 15 days of therapy, where Chi^2^ test < 0.05. SPS; sodium polystyrene sulfonate, SZC; sodium zirconium cyclosilicate, Novel K + ; Binder (SZC + patiromer)

Within the first 15 days after dispensation, mean potassium decreased to 4.6 (SD 0.6) and 4.8 (SD 0.6) mmol/L in new users of sodium polystyrene sulfonate and novel binders, respectively, a level that was maintained during the following 60 days (Table 2 and Fig. 2, panels A and B). Figure 2, panels C and D depict the change in potassium concentration from baseline levels, observing similar average potassium reduction between treatments. There were no statistically significant differences in achieved potassium levels between treatments in multivariable logistic regression that adjusted for differences in patient characteristics between groups. The OR for novel potassium binders vs. sodium polystyrene sulfonate was 0.65 (95% CI 0.38–1.10) during the first 15 days of therapy and 0.89 (95% CI 0.45–1.76) after 60 days (Table 2). In unadjusted analysis, 76% (95% CI 73- 79) of new users of sodium polystyrene sulfonate and 67% (95% CI 56–77) of new users of novel binders reached a potassium value ≤ 5.0 mmol/L after 15 days of therapy (Fig. 3). At 60 days, 73% of patients maintained potassium ≤ 5.0 mmol/L in both groups. Similar results were obtained in a sensitivity analysis defining achieved potassium concentration with the median value of all tests during each interval (Supplementary Figs. 1 and 2). SPS: sodium polystyrene sulfonate, SZC: sodium zirconium cyclosilicate.

**Table 2:** Achieved potassium levels during the first 60 days and odds ratio (OR) and 95% confidence intervals (CI) for normokalemia (potassium ≤ 5.0 mmol/L) associated with the initiation of novel potassium binders (compared to sodium polystyrene sulfonate)

Variable	SPS	Novel potassium binders	Patiromer	SZC
*n*	1879	147	41	106
1–15 days				
Patients tested for potassium, *n* (%)	1081 (58)	82 (56)	23 (56)	59 (56)
Number of potassium tests, median (IQR)	2 (1–3)	2 (1–2)	1 (1–2)	2 (1–3)
Last potassium within the interval, mean (SD)	4.6 (0.6)	4.8 (0.6)	4.8 (0.5)	4.8 (0.7)
Adjusted OR (95% CI)	REF	0.65 (0.38–1.10)	–	–
> 15–30 days				
Patients tested for potassium, *n* (%)	873 (48)	76 (52)	20 (46)	56 (56)
Number of potassium tests, median (IQR)	2 (1–3)	1 (1–2)	1 (1–2)	1 (1–3)
Last potassium within the period, mean (SD)	4.7 (0.7)	4.8 (0.6)	4.8 (0.4)	4.8 (0.6)
Adjusted OR (95% CI)	REF	0.75 (0.41–1.36)	–	–
> 30–45 days				
Patients tested for potassium, n (%)	752 (40)	54 (37)	13 (32)	41 (38)
Number of potassium tests, median (IQR)	1 (1- 3)	2 (1–2)	1 (1–2)	2 (1–3)
Last potassium within the period, mean (SD)	4.7 (0.7)	4.9 (0.8)	5.0 (0.6)	4.8 (0.9)
Adjusted OR (95% CI)	REF	0.56 (0.30–1.06)	–	–
> 45- 60 days				
Patients tested for potassium, *n* (%)	725 (39)	59 (40)	13 (32)	46 (43)
Number of potassium tests, median (IQR)	1 (1–2)	1 (1–2)	1 (1)	1 (1–2)
Last potassium within the period, mean (SD)	4.7 (0.7)	4.7 (0.6)	4.9 (0.4)	4.7 (0.7)
Adjusted OR (95% CI)	REF	0.89 (0.45–1.76)	–	–
1–60 days				
Patients with at least one K > 5.5 mmol/L, *n* (%)	365(24)	31(26)	4(13)	27(31)
Standardized potassium coefficient of variation (%)	16	14	10	15

Multivariable logistic regression adjusted by age, gender, eGFR, comorbidities (diabetes, hypertension, myocardial infarction, heart failure, arrhythmia) and medications (beta-blockers, thiazide, loop diuretics, ACEI/ARB and MRA)

*SPS* sodium polystyrene sulfonate, *SZC* sodium zirconium cyclosilicate

Within 365 days of follow-up, 3 patients had a major gastrointestinal event (gastrointestinal ulcer and/or perforation in two patients who started sodium polystyrene sulfonate and one patient who started a novel binder). The characteristics of these 3 patients are described in Supplementary Table 4. Minor adverse events are described in Table 3. The most common identified adverse event was hypocalcemia, with a cumulative incidence of 27% of patients who started sodium polystyrene sulfonate by day 90, and 19% of patients who started novel binders. In multivariable analyses, adjusted for age, gender, eGFR, diabetes, hypertension, myocardial infarction, heart failure, and arrhythmia, but not for baseline plasma calcium, the odds for hypocalcemia were numerically lower in the initiators of novel binders than sodium polystyrene sulfonate initiators, however, did not reach statistical significance [OR 0.71 (95% CI 0.44–1.15) *p* = 0.14]. The second most common adverse event detected was initiation of constipation or diarrhea medications, with a cumulative incidence of 20% in patients who started sodium polystyrene sulfonate by day 90, and 14% in patients who started novel binders (*p* = 0.17). Hypokalemia, hypophosphatemia, and hypernatremia followed the same pattern, with non-statistically significant lower multivariate odds for novel binders vs. sodium polystyrene sulfonate (Table 3**).** A description of death events during follow up is reported in Supplementary Table 5.

**Table 3:** Number of detected adverse events associated to potassium binders during the first 90 days of therapy and odds ratio (with 95% confidence intervals) for novel potassium binders compared to sodium polystyrene sulfonate (reference group)

	Observation period	1–30 days	1–60 days	1–90 days
Initiation of constipation or diarrhea medications (among non-users at index date)				
SPS (*n* = 1620)	Events (% total)	152 (9)	258 (16)	327 (20)
Novel potassium Binders (*n* = 132)	Events (% total)	7 (5)	14 (11)	19 (14)
	Adjusted OR (95% CI)	0.65 (0.30–1.45)	0.70 (0.38–1.29)	0.76 (0.45–1.28)
Hypokalemia (potassium < 3.5 mmol/L)				
SPS (*n* = 1879)	Events (% total)	110 (6)	170 (9)	208 (15)
Novel potassium Binders (*n* = 147)	Events (% total)	9 (6)	12 (8)	14 (10)
	Adjusted OR (95% CI)	0.87 (0.42–1.81)	0.79 (0.43–1.50)	0.74 (0.41–1.34)
Hypocalcemia (calcium < 2.15 mmol/L)				
SPS (n = 1879)	Events (% total)	299 (16)	413 (22)	504 (27)
Novel potassium Binders (*n* = 147)	Events (% total)	14 (10)	22 (15)	28 (19)
	Adjusted OR (95% CI)	0.75 (0.38–1.45)	0.69 (0.41–1.18)	0.71 (0.44–1.15)
Hypophosphatemia (phosphate < 0.81 mmol/L)				
SPS (n = 1879)	Events (% total)	72 (4)	99 (5)	121 (6)
Novel potassium Binders (*n* = 147)	Events (% total)	3 (2)	5 (3)	6 (4)
	Adjusted OR (95% CI)	0.58 (0.17–1.99)	0.66 (0.25–1.73)	0.64 (0.26–1.55)
Hypernatremia (sodium > 145 mmol/L)				
SPS (*n* = 1879)	Events (% total)	69 (4)	104 (6)	133 (7)
Novel potassium Binders (*n* = 147)	Events (% total)	3 (2)	4 (3)	4 (3)
	Adjusted OR (95% CI)	0.55 (0.16–1.83)	0.52 (0.18–1.45)	0.41 (0.15–1.15)

Multivariable logistic regression adjusted for age, gender, *eGFR* diabetes, hypertension, myocardial infarction, heart failure, arrhythmia

*SPS* sodium polystyrene sulfonate

## Discussion

In this study, we observed longer treatment duration for novel binders and more frequent chronic use than for sodium polystyrene sulfonate. However, all three agents showed similar short-term effectiveness in reducing plasma potassium, and similar safety with regard to minor adverse events.

The observed longer treatment duration of novel binders than for sodium polystyrene sulfonate agrees with their marketed indication for chronic use. It also agrees with observations from a German study of patients who received prescriptions for patiromer, where the majority were maintained on therapy for up to 1 year [13]. A strength of our analysis is that it is based on filled dispensations, a better method of ascertainment of exposure than prescriptions. The observed absolute and relative potassium reductions in our study are also in line with trials [6, 8, 9, 14, 32] and observational studies [13, 16–20], A summary table of all identified trials and observational evidence to date is shown in Supplementary Table 6 and 7.

A novelty in our study is the observation that all agents were associated with similar reductions in potassium levels. With one exception [10], available trial evidence to date compared single potassium binders against the non-pharmacological standard-of-care. This lack of formal comparisons precludes the ability to conclude that one agent is more or less potent than another. Although we did not find statistically significant differences in potassium levels between agents, the odds ratio for reaching normokalemia (potassium < 5.0 mmol/L) in the first 15 days of follow-up was numerically lower for novel potassium binders vs. sodium polystyrene sulfonate, and this tendency continued throughout the follow-up. A recent randomized cross-over trial [10], where 48 patients on hemodialysis were given 2-weeks of sodium polystyrene sulfonate 15 g tid or patiromer 16.8 g daily with a 2-week washout period, showed that mean weekly potassium was 4.6 mmol/L during sodium polystyrene sulfonate periods compared to 5.2 mmol/L during washout periods (difference of 0.6 mmol/L, *p* < 0.01), and compared to 5.0 mmol/L during patiromer periods (difference of 0.2 mmol/L, *p* < 0.01), suggesting higher efficacy for sodium polystyrene sulfonate than for patiromer. We note that a controlled head-to-head trial has just been initiated in the setting of acute hyperkalemia [33]. In a retrospective observational study, Huda et al. compared effectiveness in 138 patients with hyperkalemia receiving sodium zirconium cyclosilicate or calcium polystyrene sulphonate in the United Kingdom, and observed similar efficacy in promptly reducing elevated potassium levels [18]. A similar study in Japan by Nakayama et al. [33] that included 132 patients, observed however a greater reduction in potassium levels by sodium zirconium cyclosilicate compared to calcium polystyrene sulfonate. Calcium polystyrene sulfonate is not commercialized in Sweden and we cannot confirm or refute these observations.

Another novelty in this study is the observation that all agents showed similar short-term incidence of minor adverse effects. The rates of adverse events for sodium polystyrene sulfonate in our study are comparable to the two existing small trials: hypocalcemia 18.8%, hypokalemia 18.8%, nausea 25–43%, constipation 8–37.5% and anorexia 34% [9, 34]. Our observed rates are also compatible with the original Food and Drug Administration (FDA) filings for sodium zirconium cyclosilicate approval, the adverse events reported were edema (13.7%), hypertension (11%) and heart failure (4.6%) [35], with higher incidence of edema in high-risk individuals (CKD, heart failure). In the original FDA filings for patiromer approval, hypokalemia and hypomagnesemia were noted to occur in 3–10% and 5–17% based on dose, respectively [11].

We did not find any statistically significant differences in the rate of adverse effects between agents. However, absolute risks were numerically higher for sodium polystyrene sulfonate users, and the relative risks showed consistently lower odds ratios for novel binders compared to sodium polystyrene sulfonate. The broad confidence intervals are likely due to our small sample size, but results may also be impacted by perception of risk by clinicians, resulting in different monitoring rates and laboratory testing between treatment groups, thereby increasing the chance of finding abnormalities. This is different from the scenario of trials, where all patients are monitored with the same rate and frequency as per protocol [36, 37]. Finally, very few serious gastrointestinal events identified through administrative data have been reported to date for sodium polystyrene sulfonate alone, finding that small absolute risks that compared to non-pharmacological standard of care were statistically significant [23, 38] or no different [39]. Large administrative data studies on rare adverse events with novel binders are currently not available, and our study was not powered to evaluate them.

Our study has additional limitations: The study population is representative of Stockholm, which may not necessarily reflect experiences in other regions or demographic groups. In our study, we assumed standard doses and lacked information on prescribed doses. Results may thus be in part explained by the prescription of a higher effective dose rather than differences between agents at equipotent doses. No direct information on dose equivalence is available in the literature, except for the observational study by Kovesdy et al*. *[16], which found sodium polystyrene sulfonate 15 g tid more effective than patiromer 16.8 g daily. We have no information on co-interventions during the same period [such as changes in diet, stopping renin-angiotensin system inhibitors or angiotensin receptor neprilysin inhibitor, or prescribing potassium-lowering diuretics]. Our study focuses on the reduction of plasma potassium as a surrogate endpoint for preventing severe hyperkalemia and related adverse events. However, it does not directly investigate clinical outcomes such as arrhythmia or mortality, which are the ultimate endpoints of interest. We believe that a potentially slow adoption of novel binders in our region, as seen in this study, could reflect a combination of cultural and historical practices, a need for more familiarity and confidence among clinicians, and financial considerations. There is also a need for better data on relative effectiveness and for data on cost effectiveness.

To conclude, this study of new users of potassium-lowering agents from Stockholm shows similar effectiveness and adverse event rates for patients treated with sodium polystyrene sulfonate and those treated with novel binders. However, treatment duration was longer for the novel agents, which may raise questions on cost-effectiveness [40]. Moving forward, it would be important to confirm these findings through a clinical trial and evaluation of cost-effectiveness [18, 41]. In addition to preventing hyperkalemia-related arrhythmic death, it has been suggested that prolonged use of binders may prevent clinically important events by permitting continuation of the evidence-based cardiovascular medications [42], thereby reducing cardiovascular outcomes. However, these cardiovascular events are rare and numbers needed to treat, as well as duration of treatment with potassium binders to prevent one event, will likely be very high compared to other cardiovascular prophylactic strategies [43].

### Supplementary Information

Below is the link to the electronic supplementary material.

Supplementary file1 (DOCX 4872 KB)

## Data Availability

The data contain patient-related information and cannot be shared publicly as per European General Data Protection Regulation. The data can be accessed through collaborative research applications address to the principal investigator JJC (juan.jesus.carrero@ki.se), and subjected to data sharing agreements that fulfill institutional and national regulations.
