# Supplementary material for: Evaluation of the introduction of novel potassium binders in routine care; the Stockholm CREAtinine measurements (SCREAM) project
Source: J Nephrol. 2024 Jan 18;37(4):961–72. doi: 10.1007/s40620-023-01860-0 (PMC11239771; doi:10.1007/s40620-023-01860-0)
Supplement: Supplementary file 1 — Supplementary file1 (DOCX 4872 KB) [file 40620_2023_1860_MOESM1_ESM.docx]

**Supplemental Material**

**Evaluation of the introduction of novel potassium binders in routine care; the Stockholm CREAtinine Measurements (SCREAM) project**

Ailema Gonzalez-Ortiz ^1,2^, [Catherine M Clase](https://pubmed.ncbi.nlm.nih.gov/?sort=date&size=100&term=Clase+CM&cauthor_id=36280224) [^3^](https://pubmed.ncbi.nlm.nih.gov/36280224/#affiliation-8)^,4^,  [Alessandro Bosi](https://pubmed.ncbi.nlm.nih.gov/?sort=date&size=100&term=Bosi+A&cauthor_id=36459371)^1^, [Edouard L Fu](https://pubmed.ncbi.nlm.nih.gov/?sort=date&size=100&term=Fu+EL&cauthor_id=36280224) ^1,^[^5^](https://pubmed.ncbi.nlm.nih.gov/36280224/#affiliation-2)^,6^ , [Beatriz E Pérez-Guillé](https://pubmed.ncbi.nlm.nih.gov/?sort=date&size=100&term=P%C3%A9rez-Guill%C3%A9+BE&cauthor_id=36158743)^2^, Anne-Laure Faucon^1,7^, [Marie Evans](https://pubmed.ncbi.nlm.nih.gov/?sort=date&size=100&term=Evans+M&cauthor_id=29667759)^8^ , [Carmine Zoccali](https://pubmed.ncbi.nlm.nih.gov/?sort=date&size=100&term=Zoccali+C&cauthor_id=34221367)^9^,  [Juan-Jesús Carrero](https://pubmed.ncbi.nlm.nih.gov/?sort=date&size=100&term=Carrero+JJ&cauthor_id=36280224) [^1^](https://pubmed.ncbi.nlm.nih.gov/36280224/#affiliation-1)^,10^

| **Supplementary Table 1.** Definition or comorbidities…………………………………….2 |
| --- |
| **Supplementary Table 2.** Definition of medications………………………………………2 |
| **Supplementary Table 3.** Definition of adverse gastrointestinal events …………………………………………………………………………….……………….2  **Supplementary Table 4.** Characteristics of patients who presented major GI events……3  **Supplementary Table 5.** Mortality events at 3, 6 and 12 months according to treatment.3  **Supplementary Table 6.** Summary of evidence for novel potassium binders  clinical trials………………………………………………………………………..........4-6 |
| **Supplementary Table 7.** Summary of evidence for novel potassium binders  observational studies………………...…………………………….…………………….7-9 |
| **Supplemental Figure 1:** Mean potassium concentration during different intervals after treatment starts. ….…………………….………………………………………………….10 |
| **Supplementary Figure 2.** Proportion of patients with potassium concentration <5.1 and 5.5 mmol/L pre and post-potassium binder initiation ……………………….………….11 |
| **References** ………………………………………………….…………………………12-13 |

| **Supplementary Table 1.** Definition of comorbid conditions | |
| --- | --- |
| **Condition** | **ICD-10 code** |
| Diabetes mellitus | E10-14 |
| Hypertension | I10-I15 |
| Myocardial infarction | I21-22, I252 |
| Heart failure | I110, I130, I132, I50 |
| Arrhythmia | I44-I47, I49 |
| Hyperkalemia | E875 |
| Inflammatory bowel disease | K58 |

| **Supplementary Table 2**. Definition of ongoing medications | |
| --- | --- |
| **Medication** | **ATC Code** |
| Betablocker | C07 |
| Mineralocorticoid receptor antagonist (MRA) | C03DA01, C03DA04 |
| Thiazide/Loop diuretics | C03A/C03C |
| ACE inhibitor | C09A/C09B |
| ARB | C09C/C09D/C09X |
| Antiplatelets/Aspirin | B01AC |
| Proton Pump Inhibitors | A02BC |

**Supplementary Table 3. Definition of adverse gastrointestinal events**

|  | ICD 10 code |
| --- | --- |
| Severe adverse gastrointestinal event | Gastrointestinal ulcer and perforation (K25, K26, K27, K28, K631, K633), Intestinal ischemia or thrombosis (K550, K558, K559) |
| Minor adverse gastrointestinal event | Initiation of medication against constipation (A06AA, A06AB, A06AC, A06AD, A06AG) or antidiarrheal agents (A07D, A07B) |
|  |  |

**Supplementary Table 4. Characteristics of three patients who experienced a major GI events**

| **Characteristic** | **Patient 1** | **Patient 2** | **Patient 3** |
| --- | --- | --- | --- |
| Type of major GI event | Perforation of intestine (nontraumatic) | Ulcer of intestine | Perforation of intestine (nontraumatic) |
| Potassium binder used | SZC | SPS | SPS |
| Gender | male | male | male |
| Age years | 76 | 83 | 74 |
| *Comorbidities at index date* |  |  |  |
| Hypertension | yes | yes | no |
| Diabetes | yes | yes | no |
| Heart failure | no | no | no |
| Myocardial infarction | no | yes | no |
| Arrhythmia | no | yes | no |
| Inflammatory Bowel disease | no | no | no |
| *Medications 2 months prior event* |  |  |  |
| Betablockers | yes | no | yes |
| Thiazide/loop diuretics | yes | yes | no |
| ACE inhibitor | no | no | yes |
| Angiotensin Receptor Blocker | no | no | no |
| Mineralocorticoid receptor antagonist | no | no | no |
| Proton Pump Inhibitors | no | yes | no |
| Antiplatelets/Aspirin | no | yes | no |

**Supplementary Table 5. Deaths within 3, 6 and 12 months in each treatment group**

|  | SPS  n= 1879 | Patiromer  n=41 | SZC  n=106 |
| --- | --- | --- | --- |
| **All-cause deaths** |  |  |  |
| Within 3 Months | 90 (4.8%) | 1(2.5%) | 4 (3.8%) |
| Within 6 Months | 151 (8.0%) | 1 (2.5%) | 6 (5.6%) |
| Within 12 Months | 266 (14.2%) | 5 (12.2%) | 9 (8.5%) |
| **CVD-related deaths** |  |  |  |
| Within 3 Months | 62(3.3%) | 0 (0%) | 4 (3.8%) |
| Within 6 Months | 101(5.4%) | 0 (0%) | 5 (4.7%) |
| Within 12 Months | 167 (8.9 %) | 2 (4.9) | 6 (5.6%) |

| **Supplementary Table 6. Summary of evidence for novel potassium binders** | | | | | | |
| --- | --- | --- | --- | --- | --- | --- |
| **Clinical trials** | | | | | | |
| **Patiromer** | | | | | | |
| **STUDY** | **Population** | **Treatment**  **Design** | **Follow-up** | **Efficacy** | **Safety** | **Comments** |
| Pitt^1^  USA, Germany  Czech Republic, Poland, Ukraine, Russia, and Georgia.  2011 | ≥ 18 years of age had a history of chronic heart failure.  eGFR <60ml/min/1.73 m^2^ | double blind, randomized,  Placebo-controlled  n=60 patiromer  n=60 placebo | 4 weeks | patiromer difference -0.22 mmol/L, whereas placebo group had a change of +0.23 mmol/L. | hypokalemia 6%  Hypomagnesemia 24%  GI disorders 21%  Flatulence 7%, diarrhea, and constipation 5% | Patients were instructed to star spironolactone at dose of 25mg/day, after 2 weeks increased to 50mg/day |
| Weir ^2^  2014 | Patients eGFR 15 to < 60 mL/min/1.73 m^2^  18-80 years old  serum K+ 5.1 to < 6.5 mmol/l at screening  who were receiving RAAS inhibitors | Multicenter  Two phases study:  Phase 1 single-blind (n=243)  4.2g twice/daily or 8.4g twice/daily  Phase-2  Placebo-controlled single-blind, randomized.  n=107 in phase 2  n= 55 patiromer  n=52 placebo | 4 weeks treatment initial phase  Phase 2:  8-week patiromer or placebo | Mean change phase 1 was −1.01± 0.03mmol/L  At week-4 76% patients where normokalaemic.  Phase 2 median K+ change in patiromer was 0 mmol/L and 0.72 mmol/L in placebo | Phase 1: constipation 11% most common  Phase2: similar results between patiromer (47%) and placebo (50%) of any adverse event.  8% headache in placebo, For patiromer:  Nausea 4%,  Diarrhea 4%, constipation 4%, Headache 4% | Females of child-bearing potential were required to have been non-lactating, to have had a negative serum pregnancy test at screening, and to have used a highly effective form of contraception for at least 3 months before patiromer administration |
| Bakris ^3^  AMETHYST-DN  48 sites in 5 European countries  2015 | Outpatients with type 2 diabetes  eGFR 15 to 60ml/min/1.73 m^2^ and serum K level >5.0 mmol/L  Aged 30 to 80 years. | Multicenter  Phase 2, open-label, dose-ranging, randomized clinical trial 306 patients randomized. | Patiromer  4 weeks and 52-weeks  Mild hyperkalemia  8.4g/d  16.8 g/d  25.2 g/d  Moderate hyperkalemia  16.8 g/d  25.2 g/d  33.6 g/d | Mean K+ reduction at week 4 was:  mild hyperkalemia  8.4g/d= -0.35 mmol/L  16.8g/d= -0.51 mmol/L  25.2g/d= -0.55 mmol/L  moderate hyperkalemia  16.8 g/d= -0.87 mmol/L  25.2 g/d = -0.97 mmol/L  33.6 g/d= -0.92 mmol/L | Most common AE  Mild hyperkalemia  8.4g/d- diarrhea 8%  16.8g/d- worsening hypertension 9.6%  25.2g/d – hypomagnesemia 8.2%  Moderate hyperkalemia  16.8 g/d- worsening hypertension 15.4%  25.2 g/d- worsening CKD and hypomagnesemia 14.3%  33.6 g/d-worsening CKD 23.3% | All patients received RAAS inhibitors prior to and during study treatment. |
| Bushinsky^4^  USA  2015 | Patients with hyperkalemia and CKD who were receiving at least one RAASi. | Phase 1, open-label. Single-arm study  Patiromer 8.4g twice daily n=25 | 3-day run-in  Treatment period 48 hours  4 doses over 2 days | K+ reduction 0.21(0.07) mmol/L after 7h first dose.  At 48 h (14h after the last dose), a 0.75 (0.07) mmol/L mean reduction and 0.65(0.95, -0.36) mmol/L reduction from baseline. | No serious or severe AE reported.  Constipation in 8%  Hypotension in 8%  In total 28% reported at least one AE. | Mean eGFR was 3.8(SD 20.7)  And 100% with hypertension  28% with heart failure |
| Butler ^5^  DIAMOND  USA, South America,  Europe and Russia  2022 | 878 patients with HFrEF hyperkalemia or history hyperkalemia | n=439 patiromer  n=439 placebo  Phase 3  Randomized trial | Evaluated at weeks 1,2,6, 18 and every 3 months until  54 weeks | The adjusted mean change in serum K+ was +0.03 mmol/L (95% CI  –0.01, 0.07) in the patiromer group and +0.13 mmol/L (95% CI 0.09,  0.16) in the placebo group, difference of –0.10 mmol/L (95% CI –0.13, –0.07) | Patiromer  Hypokalemia 15%  Hypomagnesemia 4.3%  Diarrhea 4.3%  Vs  Placebo  Hypokalemia 10.7%  Hypomagnesemia 5%  Diarrhea 3.4% | The protocol required patients to have hyperkalemia at screening, while receiving ACEi, ARB, ARNi and/or MRA  eGFR <30ml/min/1.73 m^2^ |
| Jaques ^6^  Switzerland  2022 | 3 HD centers  48 patients on HD  ≥ 18 years of age | Crossover trial  n=25 Patiromer-SPS  n= 23 SPS-Patiromer | Patiromer 4 weeks followed 2week washout and finally SPS for 4 weeks. | Similar efficacy  The mean weekly k+ value throughout the study was 4.85±0.7 mmol/L  Under treatment, time no significant effect differences | Tolerability was higher under patiromer as compared to SPS.  GI side effects were reported in 25.4% and similar between treatments. | Patients were included in the present study (at the end of the 2-week washout period for those initially under K+ binders) if they had k+ values between 5.0 and 6.4 mmol/L |
| **Sodium zirconium cyclosilicate** | | | | | | |
| **STUDY** | **Population** | **Treatment**  **Design** | **Follow-up** | **Efficacy** | **Safety** | **Comments** |
| Zannand^7^  HARMONIZE-Global study  Japan, Russia, South Korea and Taiwan  2020 | Hyperkalemia ≥5.1 mmol/L, then received oral SZC 10g three times daily for 48h. | Two-phase, randomized, double-blind, placebo-controlled  n=102 SZC 5g n  n=102 SZC 10g  n=51 placebo | Measures on days  1-29 | Patients remaining normokalaemic on day 29  SZC 5g=58.6%  SZC 10g =77.3%  Placebo= 24%  OR; 95% CI vs placebo  SZC 5g=6.34 (2.69- 14.98)  SZC 10g=18.19 (7.16- 46.21) | Adverse events were reported by 28.3%(SZC 5g) 44.4%(SZC 10g)  and 20.0%(placebo)  Patients who received SZC 10 g reported a higher incidence of SMQ oedema (15.2%) and constipation (9.1%) than those  who received SZC 5 g (5.1% and 1.0%, respectively) | 78.3% with CKD , 64.4% with diabetes and 18.7% with heart failure |
| Tardif^8^  PRIORITIZE-HF  2022 | ≥ 18 years of age  Patients with heart failure and reduced ejection fraction  eGFR 20-59 ml/min/1.73m^2^ | International, multicenter, parallel-group, randomized, double-blind, placebo controlled  SZC n=92  Placebo n=90  182 patients | 12 weeks | At the end of treatment  SZC mean K+ 4.65 mmol/L  80.4% normokalaemic  Placebo mean K+ 4.9 mmol/L and 63.6% normokalaemic | Incidence adverse events was 47.3% in the SZC group and 52.2% in placebo group.  Most commonly reported adverse were chronic cardiac failure, chronic kidney disease, and viral upper respiratory tract infection in both groups. | Patients were required to have mild hyperkalemia or be at risk of developing hyperkalemia.  under-treated ACEi, ARB or ARNI and MRA |
| eGFR: estimated glomerular filtration rate, K+; potassium, AE; adverse events, RAAS; Renin angiotensin aldosterone system ,ACEi; Angiotensin converting enzyme inhibitors ARB; Angiotensin receptor blockers, ARNi; Angiotensin receptor and neprilysin inhibitor, MRA; Mineralocorticoid Receptor Antagonists, GI; Gastro intestinal, SMQ; standardized Medical Dictionary for Regulatory Activities query, SPS; sodium polystyrene sulfonate, HD; hemodialysis, SZC; Sodium zirconium cyclosilicate, OR; odds ratio, 95% CI; 95% confidence interval, CKD; chronic kidney disease, HFrEF; Hearth failure with reduced ejection fraction | | | | | | |

| **Supplementary Table 7. Summary of evidence for novel potassium binders** | | | | | | |
| --- | --- | --- | --- | --- | --- | --- |
| **Observational studies** | | | | | | |
| **STUDY** | **Population** | **Treatment**  **Design** | **Follow-up** | **Persistence** | **Effectiveness** | **Safety** |
| Kovesdy^9^  USA  2019 | HD patients  Index date firs order for patiromer/SPS or the first K+ ≥ 5.0 mmol/L | A retrospective cohort study using  n=527 Patiromer  n=865 SPS  n=8747 No K binder | Median follow-up 141 days | The mean SD proportion of days covered was 83% | Patiromer 1 month potassium 5.9 to 5.43 (0.47) at 2 months 5.39 (-0.56) =  After 1 month 24 % patients continued with hyperkalemia, and after 2 months 20%. | Data no available |
| Noel^10^  Canada  2019 | Adults ≥ 66 years old  77% had an eGFR less than 60mL/min/1.73m^2^ | Retrospective matched cohort study  27,704 adults  Newly SPS users | Individuals  were followed up until the study outcome, 30 days, emigration from the province, or death. | N/A | No data | Adverse GI event within 30 days  IR per 1000 person-year  28.33 (21.33-35.32)  HR 5.61(95% CI 4.34-7.26) |
| Kovesdy^11^  USA  2020 | Veterans with K+≥ 5.1 mmol/L  Median 70 years old  Patients had; HF (32%), diabetes (83%) or CKD (95%). | A retrospective cohort study among | 288 patients included.  6 months post-patiromer initiation | Patiromer %  1 month 97%  3 months 40%  6 months 25%  SPS  1 month 25%  3 months 40%  6 months 25% | Across all intervals, mean K+ change was -1.00 to -1.05 mmol/L | Data not available |
| Palo^12^  USA  2022 | Adult patients were treated for acute hyperkalemia in emergency department.  Mean K+ baseline 5.6±0.35 mmol/L | Cohort  n=881 patients with patiromer  A single dose 8.4g, 16.8g or 25.2g | 0-6, 6-12 and  12-24 hrs. | N/A | The mean reduction after 6 hours was 0.50 (SD 0.56) mmol/L and 12-24 hours 0.46 (SD 0.6) mmol/L | No data |
| Huda^13^  UK  2022 | Patients who received ZSC or CPS to manage hyperkalemia | Retrospective study of the electronic patient record system in hospital  n=65 ZSC  n=73 CPS | 3 days treatment | N/A | Reduction in potassium concentration SZC -1.24    CPS -1.17  NS | No data |
| Pecoits-Filho^14^  German  2022 | eGFR 15-60 ml/min/1.73 m^2^  starting patiromer | n=15427 patients  n=140 patients patiromer users  n=490 SPS users | 180 days | Only 5% of patients who initiated patiromer discontinued use within the first month.  most patients on patiromer do not discontinue treatment prior to 1 year after initiation. | Patiromer users K+ median 5.7(5.4 - 6.3) mmol/L after 1 month follow-up median was 5.4 | Describing adverse events  related to patiromer utilization was not an aim of this analysis and these data were not available to the authors. |
| Pinell ^15^  USA  2022 | Veterans with ESKD | n=458 patients  Cohort  patiromer utilization | 365 days post index date | Mean treatment course duration 30 (30-31 days)  Less than 10% persisted at 180 days. | Mean K+ at baseline 5.91 mmol/L to 4.94 mmol/L after 1 month,  4.89 mmol/L at 91 days  and 4.88 mmol/L at 182 days. | No data |
| Patel^16^  USA  2023 | Veterans  Outpatient dispensing  patiromer  95.1% eGFR <60ml/min/1.73m^2^ | National observational study  n=3149 veterans | 180-day post index date | Mean course duration 64 days (59-72)  The number of patients with only 1 treatment course was 155 (75.6%), 2 courses were 49 (23.9%), and 3 courses were 1 (0.5%) | After 30 days the mean potassium value was 4.93 mmol/L compared to baseline, mean K+ change -0.81 mmol/L at 30 days and the proportion of patients with a K+ value <5.1 was 40.5% at 30 days and less than 1% developed hypokalemia | The study  did not assess patiromer adverse events or reasons for patiromer discontinuation. |
| Nakayama^17^  Japan  2023 | All patients aged 18 years who have newly started ZSC or CPS  hospital  eGFR <60ml/min/1.73m2 | SZC vs CPS  Retrospective study  n=132 patients  n=48 ZSC  n=84 CPS | 4 weeks | 27.1% patients in SZC  22.6% in CPS discontinued after the follow-up point | K+ reduction  ZSC= -1.2 mmol/L  CPS= -0.8 mmol/L  Change baseline to end ZSC= 5.8 -4.5 mmol/L  CPS=5.7-4.9 mmol/L | Higher sodium in SZC no differences in others  highest common AE hypokalemia in 6.3% SZC |
| Shockey^18^  USA  2023 | Adults who received at least one dose of SZC after intra-abdominal transplant (kidney, pancreas and/or liver) | Retrospective- single-center study  10-g dose | 14 days | N/A | K+ change, mean (n=46) = -2.7 mmol/L | There were no gastrointestinal complications after the administration of SZC. The average increase in serum bicarbonate was 0.58 mEq (p = 0.41) following the initial SZC dose. |
| Qu^19^  Singapore  2023 | Hemodialysis patients all adults | Retrospective clinical audit,  42 community-based HD centers used SZC | The endpoint is defined as 31^st^ January 2023 or patients’ date of death, whichever came earlier.  2.13 ± 3.31 months | N/A | K+ change  -0.812(-0.691to -0.931) mmol/L  34% patients achieved normal K+ levels | All the AEs affected the GI tract; one patient experienced diarrhea (0.3%), one patient had abdominal pain (0.3%), and another experienced constipation (0.3%). None of the AEs necessitated.  immediate treatment or hospitalization |
| eGFR: estimated glomerular filtration rate, K+; potassium, AE; adverse events, GI; Gastrointestinal, SPS; sodium polystyrene sulfonate, CPS; Calcium polystyrene sulphonate, IR; incidence ratio, HR; hazard ratio, HF; Heart failure, ESKD; End stage kidney disease, HD; hemodialysis, SZC; Sodium zirconium cyclosilicate, SD; standard deviation, CI; confidence interval, CKD; chronic kidney disease. | | | | | | |

**Supplementary Figure 1:** Sensitivity analysis with the mean K+ concentration per patient at each interval. Mean potassium concentration during different intervals after treatment start (panels A and B) and mean K+ change from baseline K+ (panels C and D).


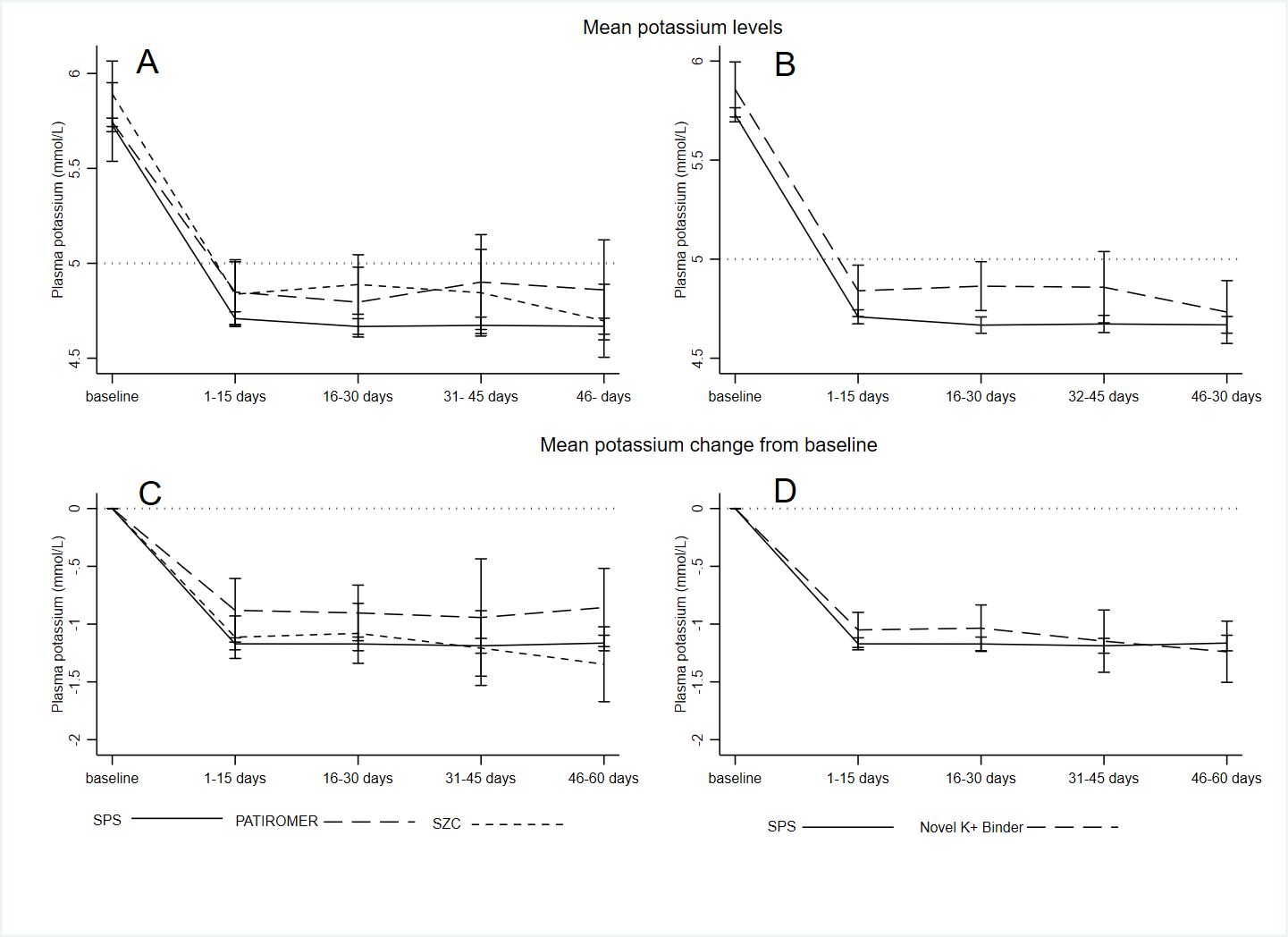


Shown is the mean K+ value per patient at each interval. Panels A and C show each agent separately (SPS, patiromer and SZC), and panels B and C combine both novel K+ binders together. There were no statistically significant differences between treatment strategies (P>0.05) as estimated by t-test or one-way ANOVA. SPS;sodium polystyrene sulfonate, SZC;sodium zirconium cyclosilicate

**Supplementary Figure 2**. Sensitivity analysis with the mean potassium concentration during different intervals after treatment start. Proportion of patients with plasma potassium concentration ≤5.0 mmol/L (Panels A and C) and ≤5.5 mmol/L (Panels B and D) before and after initiation of treatment with potassium binders.


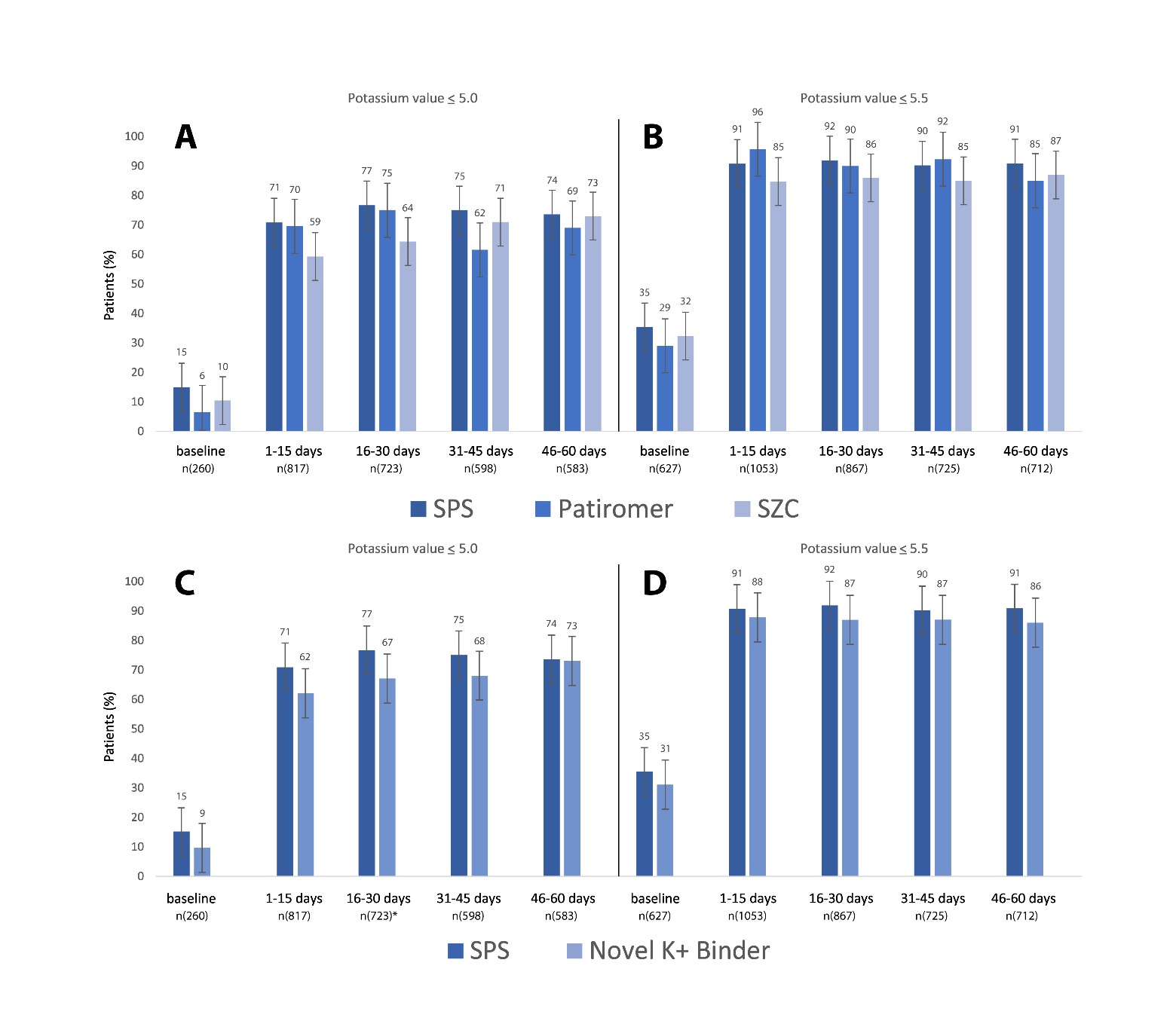


We modelled the mean K+ values per patient between each follow-up interval. N () shows the number of patients represented in each of the bars. *There were no statistically significant differences between the binders, except for one observation in panel B during the 16-30 days of therapy, where Chi^2^ test <0.05. Bars (standard error) represent the percentage of patients with potassium value ≤5.0 mmol/L and ≤5.5 mmol/L. SPS; sodium polystyrene sulfonate, SZC; sodium zirconium cyclosilicate, Novel K+; Binder (SZC+ patiromer).

**References**

1 Pitt, B. *et al.* Evaluation of the efficacy and safety of RLY5016, a polymeric potassium binder, in a double-blind, placebo-controlled study in patients with chronic heart failure (the PEARL-HF) trial. *Eur Heart J* **32**, 820-828, doi:10.1093/eurheartj/ehq502 (2011).

2 Weir, M. R. *et al.* Patiromer in patients with kidney disease and hyperkalemia receiving RAAS inhibitors. *The New England journal of medicine* **372**, 211-221, doi:10.1056/NEJMoa1410853 (2015).

3 Bakris, G. L. *et al.* Effect of Patiromer on Serum Potassium Level in Patients With Hyperkalemia and Diabetic Kidney Disease: The AMETHYST-DN Randomized Clinical Trial. *Jama* **314**, 151-161, doi:10.1001/jama.2015.7446 (2015).

4 Bushinsky, D. A. *et al.* Patiromer induces rapid and sustained potassium lowering in patients with chronic kidney disease and hyperkalemia. *Kidney Int* **88**, 1427-1433, doi:10.1038/ki.2015.270 (2015).

5 Butler, J. *et al.* Patiromer for the management of hyperkalemia in heart failure with reduced ejection fraction: the DIAMOND trial. *Eur Heart J*, doi:10.1093/eurheartj/ehac401 (2022).

6 Jaques, D. A. *et al.* Comparative efficacy of patiromer and sodium polystyrene sulfonate on potassium levels in chronic haemodialysis patients: a randomized crossover trial. *Clin Kidney J* **15**, 1908-1914, doi:10.1093/ckj/sfac129 (2022).

7 Zannad, F. *et al.* Efficacy and safety of sodium zirconium cyclosilicate for hyperkalaemia: the randomized, placebo-controlled HARMONIZE-Global study. *ESC Heart Fail* **7**, 54-64, doi:10.1002/ehf2.12561 (2020).

8 Tardif, J. C. *et al.* Potassium reduction with sodium zirconium cyclosilicate in patients with heart failure. *ESC Heart Fail*, doi:10.1002/ehf2.14268 (2022).

9 Kovesdy, C. P. *et al.* Real-World Evaluation of Patiromer for the Treatment of Hyperkalemia in Hemodialysis Patients. *Kidney international reports* **4**, 301-309, doi:10.1016/j.ekir.2018.10.020 (2019).

10 Noel, J. A. *et al.* Risk of Hospitalization for Serious Adverse Gastrointestinal Events Associated With Sodium Polystyrene Sulfonate Use in Patients of Advanced Age. *JAMA Intern Med* **179**, 1025-1033, doi:10.1001/jamainternmed.2019.0631 (2019).

11 Kovesdy, C. P. *et al.* Real-world management of hyperkalemia with patiromer among United States Veterans. *Postgrad Med* **132**, 176-183, doi:10.1080/00325481.2019.1706920 (2020).

12 Di Palo, K. E., Sinnett, M. J. & Goriacko, P. Assessment of Patiromer Monotherapy for Hyperkalemia in an Acute Care Setting. *JAMA Netw Open* **5**, e2145236, doi:10.1001/jamanetworkopen.2021.45236 (2022).

13 Huda, A. B., Langford, C., Lake, J. & Langford, N. Hyperkalaemia and potassium binders: Retrospective observational analysis looking at the efficacy and cost effectiveness of calcium polystyrene sulfonate and sodium zirconium cyclosilicate. *J Clin Pharm Ther* **47**, 2170-2175, doi:10.1111/jcpt.13766 (2022).

14 Pecoits-Filho, R. *et al.* Patiromer utilization in patients with advanced chronic kidney disease under nephrology care in Germany. *Clin Kidney J* **16**, 176-183, doi:10.1093/ckj/sfac209 (2023).

15 Pinnell, D. *et al.* Real-world evaluation of patiromer utilization and its effects on serum potassium in veterans with end stage kidney disease. *Medicine (Baltimore)* **101**, e32367, doi:10.1097/md.0000000000032367 (2022).

16 Patel, S. *et al.* Assessing patiromer utilization and associated serum potassium changes in US veterans with prior sodium polystyrene sulfonate exposure. *Medicine (Baltimore)* **102**, e33134, doi:10.1097/md.0000000000033134 (2023).

17 Nakayama, T. *et al.* Compared effectiveness of sodium zirconium cyclosilicate and calcium polystyrene sulfonate on hyperkalemia in patients with chronic kidney disease. *Frontiers in Medicine* **10**, 1137981, doi:10.3389/fmed.2023.1137981 (2023).

18 Shockey, W. *et al.* Potassium-lowering effects of sodium-zirconium cyclosilicate in the early post-transplant period. *Clin Transplant*, e15156, doi:10.1111/ctr.15156 (2023).

19 Qu, X., Hua, Y. & Khan, B. A. A Real-World Experience of Hyperkalemia Management Using Sodium Zirconium Cyclosilicate in Chronic Hemodialysis: A Multicenter Clinical Audit. *Cureus* **15**, e45058, doi:10.7759/cureus.45058 (2023).
